# Supplementary material for: Water filtration using softwood membranes provides a nature-based solution for nanoplastic removal
Source: Commun Earth Environ. 2026 Apr 4;7(1):475. doi: 10.1038/s43247-026-03469-0 (PMC13229929; doi:10.1038/s43247-026-03469-0)
Supplement: Supplementary file 2 — Supporting Information [file 43247_2026_3469_MOESM2_ESM.pdf]

Supplementary Information for
Water filtration using softwood membranes provides a nature-based solution for nanoplastic removal

Alice Pradel[1,*], Maximilian Ritter[2,3,*], Wenqing Yan[2], Denise M. Mitrano[1]

[1]Environmental Systems Science Department, ETH Zurich, 8092 Zurich, Switzerland
[2]Institute for Building Materials, ETH Zürich, 8093 Zürich, Switzerland.
[3]Cellulose and Wood Materials, Swiss Federal Laboratories for Materials Science and Technology, 8600 Dübendorf, Switzerland
[*]These authors contributed equally: Alice Pradel, Maximilian Ritter

Content

Supplementary Section 1: Pre-concentration of Pd-NPs by flocculation……...…..…...………p.2

Supplementary Figure 1: Scanning Electron Microscopy (SEM) micrographs of Pd-NPs deposited on a Si wafer……………………………………………………………………….p.3

Supplementary Figure 2: SEM micrographs of an unused spruce membrane, where no deposition of Pd-NPs can be seen either in the vicinity of the pits, nor elsewhere in the cell wall..……….p.3

Supplementary Figure 3: SEM micrographs of Pd-NPs deposition and aggregation…...………p.4

Supplementary Figure 4: SEM micrographs of a 10 mm spruce filter highlighting the accumulation of Pd-NPs in the vicinity of pits……………..……………………..…………p.5

Supplementary Section 1: Pre-concentration of Pd-NPs by flocculation.

Pd-NPs were pre-concentrated prior to digestion by flocculation. Centrifugation tubes with a capacity of 50 mL were filled with a maximum volume of 40 mL of each sample, and the mass of each sample was measured. After settling overnight, a polyacrylamide co-acrylic acid (PAM) solution (100 g L^{-1}, pH 9, Sigma-Aldrich) and aluminum nitrate nonahydrate solution (67.093 g L^{-1} of $Al(NO_3)_3$ $9H_2O$) were added to achieve concentrations of 2 800 mg L^{-1} PAM and 1 400 mg L^{-1} Al in each tube. The samples were mixed for 1.5 hours with an end-over-end shaker (Level 7 out of 10), allowed to settle overnight and then centrifuged at 10 000 g for 2 hours using an Allegra X-30R centrifuge (Beckman Coutler). After centrifugation, the supernatant was removed and the flocs the residual 200 µL at the bottom of the tube were retained for further analysis. To transfer the flocs into the microwave digestion tubes, each centrifugation tube was rinsed with 0.575 mL of distilled HNO_3 at 65% (puriss. Sigma-Aldrich). This rinsing procedure was repeated a total of four times to ensure a complete and quantitative transfer. Recovery tests were performed with ASW with the same salinity and NOM content as the underlying liquid, the ice and the brine and showed an overall recovery rate of 95 ± 5 %.

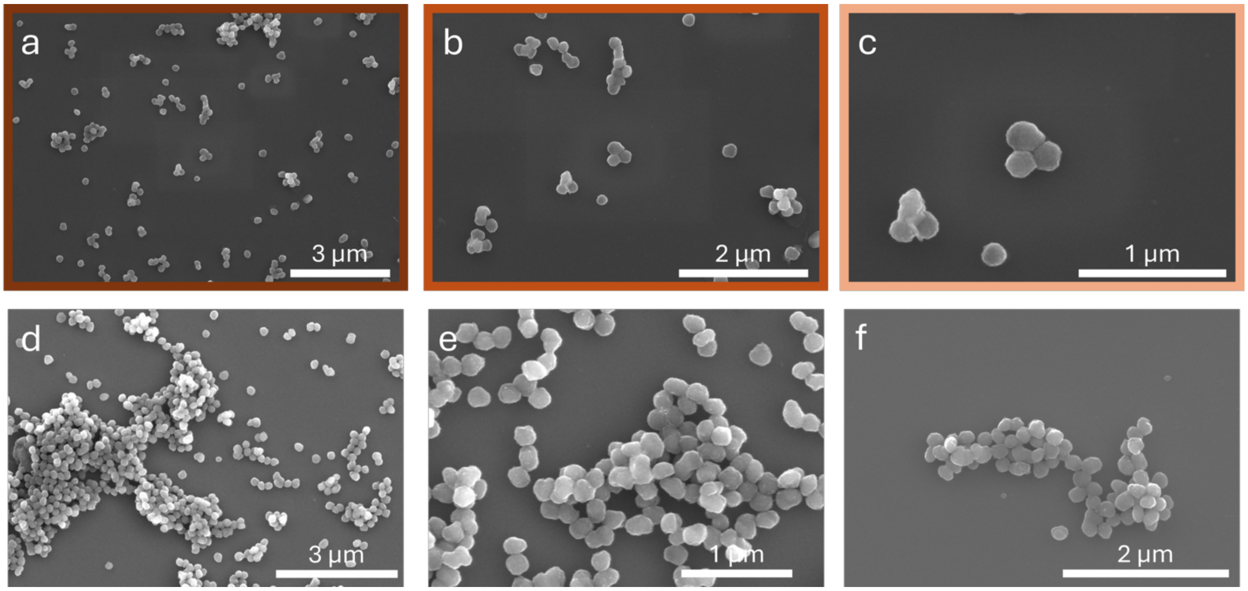

Figure S1. SEM micrographs of Pd-NPs deposited on a Si wafer. **a**) Overview of Pd-NP. **b**) magnification of central region of micrograph. **c**) Magnification of central region of b). **d**) Overview of Pd-NP. **e**) Magnification of central region of micrograph d). **f**) Magnification of central region of micrograph e). The morphology matches those of the particles found in the wood membranes.

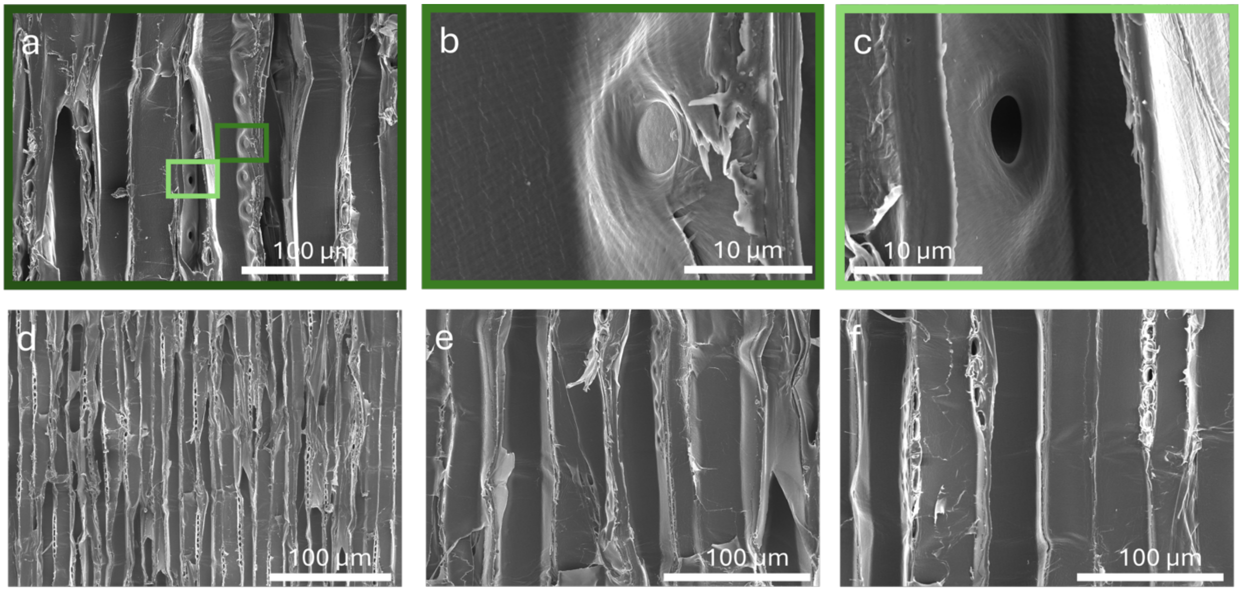

Figure S2. a) SEM micrographs of an unused spruce membrane, where no deposition of Pd-NPs can be seen, either in the vicinity of the pits, nor elsewhere in the cell wall. **b**) Region of interest of spruce membrane shown in the dark green rectangle in a). **c**) Region of interest of spruce membrane shown in the light green rectangle in a). **d**) Global view of the spruce membrane. **e-f**) Several spruce tracheids.

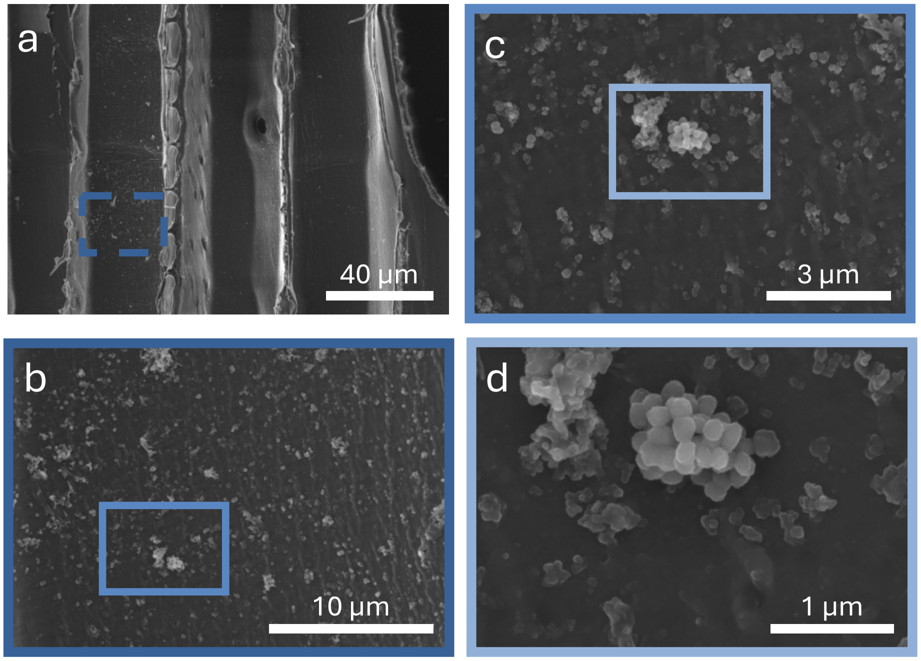

Figure S3. **a**) SEM micrograph as shown in Figure 2d. **b**) Micrographs showing the deposition of Pd-NPs on the spruce cell wall in the region of interest selected in the dashed rectangle of a). **c**) Aggregated, deposited Pd-NPs from the region of interest selected in the rectangle shown in b). **d**) Aggregated, deposited Pd-NPs from the region of interest selected in the rectangle shown in c).

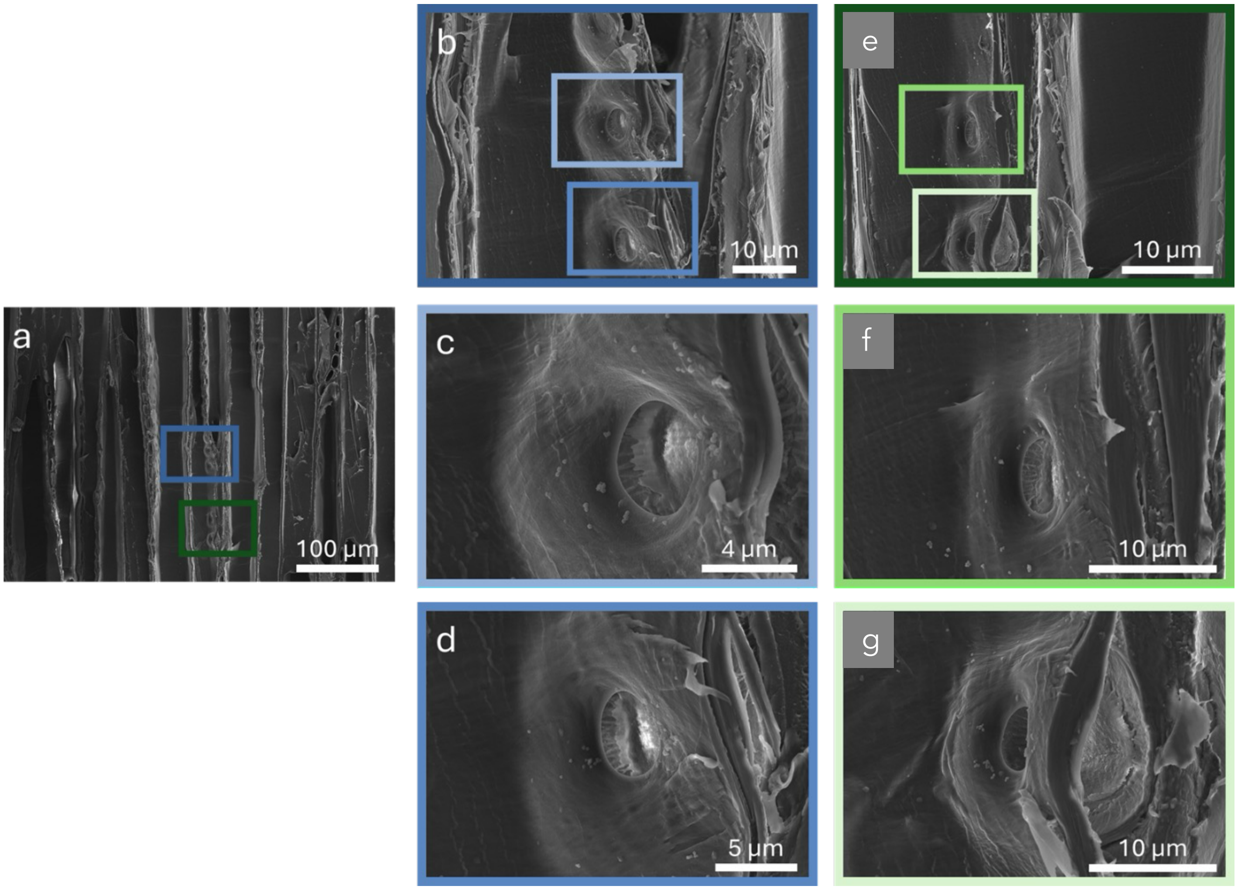

Figure S4. SEM micrographs of a 10 mm spruce filter highlighting the accumulation of NPs in the vicinity of pits. **a**) Overview of spruce xylem. **b**) Magnified view of region in blue rectangle of a). **c**) Magnified view of region in dark-blue rectangle of b). **d**) Magnified view of region in light-blue rectangle of b). **e**) Magnified view of region in green rectangle of a). **f**) Magnified view of region in dark-green rectangle of e). **g)** Magnified view of region in light-green rectangle of e).
